# Prospective assessment of loss to follow‐up: incidence and associated factors in a cohort of HIV‐positive adults in rural Tanzania

**DOI:** 10.1002/jia2.25460

**Published:** 2020-03-03

**Authors:** Aneth V Kalinjuma, Tracy R Glass, Maja Weisser, Selarine J Myeya, Bryson Kasuga, Yassin Kisung'a, George Sikalengo, Andrew Katende, Manuel Battegay, Fiona Vanobberghen, on behalf of the KIULARCO Study Group, Aschola Asantiel, Farida Bani, Manuel Battegay, Theonestina Byakuzana, Adolphina Chale, Anna Eichenberger, Sauli J Epimack, Gideon Francis, Hansjakob Furrer, Anna Gamell, Tracy R Glass, Speciosa Hwaya, Bryson Kasuga, Namvua Kimera, Andrew Katende, Yassin Kisunga, Aneth V Kalinjuma, Thomas Klimkait, Emilio Letang, Ezekiel Luoga, Lameck B Luwanda, Herry Mapesi, Ngisi P Masawa, Mengi Mkulila, Julius Mkumbo, Margareth Mkusa, Dorcas K Mnzava, Gertrud J Mollel, Germana Mossad, Lilian Moshi, Dolores Mpundunga, Athumani Mtandanguo, Selerine Myeya, Sanula Nahota, Regina Ndaki, Robert C Ndege, Agatha Ngulukila, Alex J Ntamatungiro, Amina Nyuri, Daniel H Paris, Omary N Rajab, Leila Samson, George Sikalengo, Fiona Vanobberghen, Maja Weisser, John Wigay

**Affiliations:** ^1^ Ifakara Health Institute Ifakara Tanzania; ^2^ Swiss Tropical and Public Health Institute Basel Switzerland; ^3^ University of Basel Basel Switzerland; ^4^ Department of Infectious Diseases and Hospital Epidemiology Department of Clinical Research University Hospital Basel Basel Switzerland; ^5^ USAID Boresha Afya Morogoro Tanzania

**Keywords:** lost to follow‐up, recurrent events, HIV infections, Tanzania, cohort, proportional hazards models

## Abstract

**Introduction:**

Lifelong antiretroviral therapy (ART) improves health outcomes for HIV‐positive individuals, but is jeopardized by irregular clinic attendance and hence poor adherence. Loss to follow‐up (LTFU) is typically defined retrospectively but this may lead to biased inferences. We assessed incidence of and factors associated with LTFU, prospectively and accounting for recurrent LTFU episodes, in the Kilombero and Ulanga Antiretroviral Cohort (KIULARCO) of HIV‐positive persons in rural Tanzania.

**Methods:**

We included adults (≥15 years) enrolled in 2005 to 2016, regardless of ART status, with follow‐up through April 2017. LTFU was defined as >60 days late for a scheduled appointment. Participants could experience multiple LTFU episodes. We performed analyses based on the first (prospective) and last (retrospective) events observed during follow‐up, and accounting for recurrent LTFU episodes. Time to LTFU was estimated using cumulative incidence functions. We assessed factors associated with LTFU using cause‐specific proportional hazards, marginal means/rates, and Prentice, Williams and Peterson models.

**Results:**

Among 8087 participants (65% female, 60% aged ≥35 years, 42% WHO stage 3/4, and 47% CD4 count <200 cells/mm^3^), there were 8140 LTFU episodes, after which there were 2483 (31%) returns to care. One‐year LTFU probabilities were 0.41 (95% confidence interval 0.40, 0.42) and 0.21 (0.20, 0.22) considering the first and last events respectively. Factors associated with LTFU were broadly consistent across different models: being male, younger age, never married, living far from the clinic, not having an HIV‐positive partner, lower BMI, advanced WHO stage, not having tuberculosis, and shorter time since ART initiation. Associations between LTFU and pregnancy, CD4 count, and enrolment year depended on the analysis approach.

**Conclusions:**

LTFU episodes were common and prompt tracing efforts are urgently needed. We identified socio‐demographic and clinical characteristics associated with LTFU that can be used to target tracing efforts and to help inform the design of appropriate interventions. Incidence of and risk factors for LTFU differed based on the LTFU definition applied, highlighting the importance of appropriately accounting for recurrent LTFU episodes. We recommend using a prospective definition of LTFU combined with recurrent event analyses in cohorts where repeated interruptions in care are common.

## 1 Introduction

Lifelong antiretroviral therapy (ART) is crucial to optimize health outcomes for people living with HIV 1. Retention in care is a critical component to reaching the second and third UNAIDS 90‐90‐90 targets, that is, that 90% of HIV‐positive persons are on ART, and 90% of those have suppressed viral load 2. In 2012, loss to follow‐up (LTFU) was declared as one of the key challenges for the next decade facing HIV care and treatment programmes in resource‐limited settings 3. LTFU from such programmes occurs at all steps of the care cascade, from diagnosis, during ART eligibility assessment, and after ART initiation 4, 5, 6. Irregular clinic attendance and poor ART adherence increase the risks of drug resistance, morbidity, mortality and HIV transmission 4, 5, 7, 8.

The risk of LTFU varies between HIV programmes, with estimates ranging from 0.3% to 50% 9, partly due to different time periods being considered. Furthermore, the definition of LTFU is not standardised and can have a large impact on estimates 10. The definition of LTFU should be constructed appropriately based on the research question of interest 11. Retrospective or “last event” definitions of LTFU – i.e. based on whether an individual was considered to be in care at the time of database closure – may lead to biased estimates for LTFU trends due to transient interruptions in care 11. Prospective or “first event” definitions of LTFU – that is based on first interruptions in care – are therefore preferable. Alternatively, recurrent interruptions in care could be assessed directly, thus making more efficient use of the data 12.

Previous studies have identified the factors associated with LTFU among persons on ART to include younger age, male sex, single, divorced or separated marital status, illiteracy, having no income‐generating occupation, non‐disclosure of HIV diagnosis, stigma, distance to health facilities, poor nutrition, normal body mass index, pregnancy, high or low CD4 count, tuberculosis co‐infection, advanced clinical staging, detectable viral load and adverse drug reactions 5, 8, 9, 13, 14, 15, 16, 17, 18, 19, 20, 21. However, most of these studies employed retrospective LTFU definitions; to our knowledge, no studies have assessed factors associated with recurrent LTFU episodes from HIV programmes.

Our objectives were to identify the incidence of and factors associated with LTFU among HIV‐positive adults (regardless of ART status) enrolled in the Kilombero and Ulanga Antiretroviral Cohort (KIULARCO) in a rural HIV clinic in Ifakara, Morogoro, Tanzania, prospectively and appropriately accounting for recurrent LTFU episodes.

## 2 Methods

### 2.1 Study site, population and study design

The Chronic Disease Clinic of Ifakara (CDCI) is a rural HIV care and treatment centre established in 2004, operating under Saint Francis Referral Hospital and serving people living with HIV from Kilombero and Ulanga districts in the Morogoro region in south‐east Tanzania. Kilombero and Ulanga districts have a population of approximately 700,000 and around 6% of these are people living with HIV 22. The main economic activity is rice farming, with other sources of income including fishing and mining 22. The clinic has a cohort, KIULARCO, as described previously 22, 23. Comprehensive data are systematically captured in electronic medical records, including demographic characteristics, ART use, laboratory parameters, and clinical outcomes (since 2013; previously, limited data were captured on paper). In this study, we included HIV‐positive adults (aged ≥15 years) enrolled in 2005 to 2016 with follow‐up through April 2017. We excluded transit patients (those enrolled in other clinics who attended the CDCI temporarily, usually for ART refills or a clinical consultation).

### 2.2 Outcomes

Time was measured from cohort enrolment to outcomes of death, transfer to another clinic, LTFU or in active care. LTFU was defined as >60 days late for the next scheduled appointment 24, with visits scheduled every three months for those on ART and every six months for those not yet on ART. Participants could experience multiple LTFU episodes after returning to care in the interim. Analyses were performed (a) based on the first event observed for each participant during follow‐up (prospectively) and (b) accounting for recurrent LTFU episodes, that is, participants could contribute multiple LTFU episodes if they had transient interruptions in care, with appropriate statistical methods to account for the correlations caused by multiple events within the same participant. For comparison purposes, we also performed analyses based on the last event observed before database closure regardless of previous event history (retrospectively), since this definition is commonly used in the literature. The number of participants was the same in all analyses.

### 2.3 Covariates

We included covariates based on prior knowledge of potentially being associated with LTFU. Baseline covariates were sex, age, highest education level, occupation, marital status, distance of residence from the clinic (estimated based on centre of ward of residence), partner HIV status, disclosure of HIV status, pregnant, body mass index (BMI) 25, CD4 cell count, HIV WHO stage, tuberculosis status (positive if positive microscopy with acid‐fast bacilli, positive Xpert MTB/RIF assay (Cepheid, Sunnyvale, CA, USA) in sputum or other extra‐pulmonary sample, chest radiograph suggestive of tuberculosis plus at least one symptom, physician diagnosis by ICD‐10 code, or prescription of anti‐tuberculosis medication), and enrolment year 23. Participants were considered on ART at baseline if they initiated before or within 30 days of enrolment. CD4 count, BMI, WHO stage and tuberculosis diagnosis were those measurements closest to enrolment, provided within 30 days. We incorporated time‐dependent variables of pregnancy (captured as binary variable, that is, pregnant or not, during clinical visits) and time since ART initiation during follow‐up (not yet initiated, or initiated <6, 6 to <12, 12 to <24 or ≥24 months ago). Guidelines for the management of HIV/AIDS changed during the study: in 2005 to 2011, ART was recommended for adults and adolescents with advanced HIV/AIDS 26, 27; from 2012, those with WHO stage 3/4 and CD4 count <350 cells/mm^3^ were eligible 28; from 2015, ART was available for those with CD4 count <500 cells/mm^3^ or co‐infections such as tuberculosis 29.

### 2.4 Statistical methods

#### 2.4.1 First and last event

In the presence of competing risk events (deaths and transfers), we analysed time to first LTFU event using cumulative incidence functions 30, 31. Cause‐specific proportional hazards models were used to assess factors associated with time to first LTFU episode, with deaths and transfers censored 31, 32. We considered univariable and multivariable models, excluding education and occupation which were missing for >40% of participants since they were not systematically captured until 2013. The same methods were used for the last event analysis.

We used multiple imputation with chained equations to address missing baseline covariates, assuming data were missing at random 33. In the imputations, we used predictive mean matching for square‐root‐transformed BMI and CD4 count. We used logistic regression for the binary variables and multinomial regression for the remaining categorical variables. In addition to the baseline covariates, we included in the imputations time since ART initiation over all follow‐up, whether ever pregnant during follow‐up, an indicator for LTFU for the first event, and the Nelson‐Aalen estimator of the baseline cumulative hazard 33, 34. We used 20 imputations, based on the approximate fraction of missing information 35.

#### 2.4.2 Recurrent LTFU events

Participants often experienced multiple LTFU episodes; such data require specialized approaches, because recurrent events in the same participant are not independent 36, 37. We used marginal means/rates models 38 and Prentice, Williams and Peterson (PWP) 39 models. Marginal means/rates models characterize the mean or rate of the event counting process within individuals, without specifying a covariance structure among recurrent events 12. PWP models are extensions to Cox models and correct the variance to account for within‐person correlations, with only individuals who have experienced the (k‐1)^th^ event being at risk for the k^th^ event 12, 34. We fitted PWP models with total time and gap time, with the number of LTFU events truncated at four to avoid unstable parameter estimates 12. The PWP‐total and ‐gap models differ in the risk intervals, with the former measuring time from enrolment to the event, whereas the latter resets the clock after each event and measures time between successive events 12, 34. In all models, participants who died or transferred out were censored at the time of those events. Participants were not considered at risk during a LTFU episode, but could re‐enter the risk set if they returned to the clinic (similarly following transfers). We also performed multiple imputation in a similar way, except that we included in addition LTFU indicators and baseline cumulative hazards for up to the fourth LTFU event 34.

Post‐hoc, we assessed interactions between CD4 count and enrolment year due to changes in treatment guidelines over time. Analyses were performed using Stata 40.

### 2.5 Ethical considerations

Ethical approval for the KIULARCO cohort has been obtained from Ifakara Health Institute Review Board and the National Health Research Committee of the National Institute of Medical Research of Tanzania. Written informed consent is sought from all participants at registration at the CDCI; those who refused consent were excluded.

## 3 Results

Of the 9251 participants enrolled in 2005 to 2016, we excluded 795 aged <15 years, eight with missing birth date, and 361 transit patients. We therefore included 8087 adults: the majority were female (65%), aged ≥35 years (60%), had no or only primary education (94%), were farmers (86%), were married or cohabiting (55%), lived in Ifakara town (44%), had a partner with unknown HIV status (67%), and had disclosed their HIV status (68%; Table 1). 6% of women were pregnant. Large proportions of participants had normal BMI (60%), had CD4 count <200 cells/mm^3^ (47%), were WHO stage 1/2 (58%), did not have tuberculosis (93%), and did not initiate ART within 30 days of enrolment (54%). HIV disclosure, BMI and CD4 count had high missingness, mainly from earlier calendar years. Excluding education and occupation (not captured before 2013), 14% of baseline covariates were missing across all participants.

**Table 1:** Baseline characteristics by participant outcome (based on first event to occur during follow‐up)

Characteristic	In care	Lost to follow‐up	Died	Transferred to another clinic	Total
Total, number (row %)	1781 (22%)	5105 (63%)	516 (6%)	685 (8%)	8087 (100%)
Sex					
Male	555 (31%)	1841 (36%)	212 (41%)	240 (35%)	2848 (35%)
Female	1226 (69%)	3249 (64%)	304 (59%)	441 (65%)	5220 (65%)
Missing	0	15 (<1%)	0	4 (1%)	19 (<1%)
Age, years					
15 to 24	115 (6%)	418 (8%)	29 (6%)	61 (9%)	623 (8%)
25 to 34	515 (29%)	1723 (34%)	153 (30%)	201 (29%)	2592 (32%)
35 to 44	627 (35%)	1751 (34%)	183 (35%)	244 (36%)	2805 (35%)
≥45	524 (29%)	1213 (24%)	151 (29%)	179 (26%)	2067 (26%)
Highest education level					
None	155 (9%)	191 (8%)	13 (11%)	37 (11%)	396 (9%)
Primary school	1501 (85%)	2050 (86%)	103 (86%)	289 (84%)	3943 (85%)
Secondary school and above/other	116 (7%)	148 (6%)	4 (3%)	20 (6%)	288 (6%)
Missing	9 (1%)	2716 (53%)	396 (77%)	339 (49%)	3460 (43%)
Occupation					
Farmer	1526 (86%)	2038 (85%)	105 (88%)	303 (88%)	3972 (86%)
Non‐farmers	246 (14%)	351 (15%)	15 (13%)	43 (12%)	655 (14%)
Missing	9 (1%)	2716 (53%)	396 (77%)	339 (49%)	3460 (43%)
Marital status					
Married/cohabiting	1042 (59%)	2671 (53%)	219 (46%)	395 (60%)	4327 (55%)
Never married	149 (8%)	842 (17%)	121 (25%)	73 (11%)	1185 (15%)
Separated/divorced/widowed	584 (33%)	1485 (30%)	141 (29%)	191 (29%)	2401 (30%)
Missing	6 (<1%)	107 (2%)	35 (7%)	26 (4%)	174 (2%)
Distance of residence from clinic, km					
≤1 (i.e. resident in Ifakara town)	800 (46%)	1935 (43%)	240 (55%)	176 (31%)	3151 (44%)
2 to <50	608 (35%)	984 (22%)	63 (15%)	127 (23%)	1782 (25%)
50 to <80	98 (6%)	605 (13%)	62 (14%)	60 (11%)	825 (11%)
≥80	244 (14%)	960 (21%)	68 (16%)	196 (35%)	1468 (20%)
Missing	31 (2%)	621 (12%)	83 (16%)	126 (18%)	861 (11%)
Partner HIV sero‐status					
Positive	329 (20%)	636 (13%)	36 (7%)	116 (18%)	1117 (14%)
Negative	163 (10%)	338 (7%)	28 (6%)	48 (7%)	577 (7%)
Unknown	784 (47%)	3605 (73%)	385 (78%)	405 (62%)	5179 (67%)
Not applicable	389 (23%)	340 (7%)	47 (9%)	88 (13%)	864 (11%)
Missing	116 (7%)	186 (4%)	20 (4%)	28 (4%)	350 (4%)
HIV status disclosure					
Not disclosed	410 (26%)	1486 (35%)	76 (23%)	138 (25%)	2110 (32%)
Disclosed	1176 (74%)	2736 (65%)	257 (77%)	415 (75%)	4584 (68%)
Missing	195 (11%)	883 (18%)	183 (35%)	132 (19%)	1393 (17%)
Pregnant^a^					
No	1128 (92%)	3066 (94%)	297 (98%)	417 (95%)	4908 (94%)
Yes	98 (8%)	183 (6%)	7 (2%)	24 (5%)	312 (6%)
BMI, kg/m^2^ b					
Underweight (<18.5)	322 (20%)	1075 (27%)	154 (42%)	154 (28%)	1705 (26%)
Normal (18.5 to <25)	984 (62%)	2397 (60%)	186 (51%)	335 (60%)	3902 (60%)
Overweight (≥25)	292 (18%)	546 (14%)	23 (6%)	66 (12%)	927 (14%)
Missing	85 (5%)	904 (18%)	146 (29%)	106 (16%)	1241 (16%)
CD4 count, cells/mm^3^					
<100	335 (25%)	741 (25%)	133 (53%)	126 (29%)	1335 (27%)
100 to 199	290 (21%)	554 (19%)	38 (15%)	104 (24%)	986 (20%)
200 to 349	352 (26%)	609 (21%)	34 (14%)	97 (22%)	1092 (22%)
350 to 499	190 (14%)	429 (15%)	29 (12%)	54 (12%)	702 (14%)
≥500	191 (14%)	585 (20%)	17 (7%)	58 (13%)	851 (17%)
Missing	423 (24%)	2187 (43%)	265 (51%)	246 (36%)	3121 (39%)
WHO stage					
Stage 1	693 (41%)	1988 (40%)	79 (16%)	229 (34%)	2989 (38%)
Stage 2	364 (22%)	1019 (21%)	71 (14%)	126 (19%)	1580 (20%)
Stage 3	471 (28%)	1299 (26%)	166 (34%)	215 (32%)	2151 (28%)
Stage 4	156 (9%)	618 (13%)	175 (36%)	99 (15%)	1048 (13%)
Missing	97 (5%)	181 (4%)	25 (5%)	16 (2%)	319 (4%)
Tuberculosis status					
No	1488 (90%)	4747 (95%)	474 (92%)	603 (90%)	7312 (93%)
Yes	164 (10%)	243 (5%)	41 (8%)	65 (10%)	513 (7%)
Missing	129 (7%)	115 (2%)	1 (<1%)	17 (2%)	262 (3%)
ART status (within 30 days of enrolment)					
Not yet initiated ART	530 (30%)	3172 (62%)	333 (65%)	349 (51%)	4384 (54%)
Initiated ART	1251 (70%)	1933 (38%)	183 (35%)	336 (49%)	3703 (46%)
Year of registration					
2005 to 2007	121 (7%)	1312 (26%)	224 (43%)	143 (21%)	1800 (22%)
2008 to 2009	208 (12%)	1776 (35%)	131 (25%)	176 (26%)	2291 (28%)
2010 to 2012	344 (19%)	1069 (21%)	58 (11%)	116 (17%)	1587 (20%)
2013 to 2014	417 (23%)	461 (9%)	22 (4%)	93 (14%)	993 (12%)
2015 to 2016	691 (39%)	487 (10%)	81 (16%)	157 (23%)	1416 (18%)

Results are number and column % of those with non‐missing data, unless otherwise indicated; missing data rows are number and column %. Missing data for sex, education, occupation and marital status happened before 2013. Missing data for distance, partner HIV sero‐status, HIV disclosure, BMI, CD4 count and WHO stage occurred mainly before 2013. Tuberculosis status was most frequently missing from 2013 onwards. ART, antiretroviral therapy; BMI, body mass index.

a Percentages are of number of women;

b Excluding pregnant women.

### 3.1 First event

Considering the time to the first event only, 5105 (63%) participants were LTFU, while 516 (6%) died and 685 (8%) transferred out (Table 1). There were some differences in the distribution of baseline characteristics by event, most notably those living far from the clinic being more likely to be LTFU or transfer out. Participants without partners or partners with known HIV status, and those who initiated ART at baseline, were more likely to remain in care. Participants classified as underweight, with low CD4 count or high WHO stage were more likely to die.

By the time of the first event, there had been 1001 pregnancies in 786 (15%) women. At the time of the first event, most participants in active care or who had transferred out had initiated ART (97% and 71% respectively), whereas smaller proportions of those LTFU or who had died had initiated ART (57% and 52% respectively).

**Figure 1:**
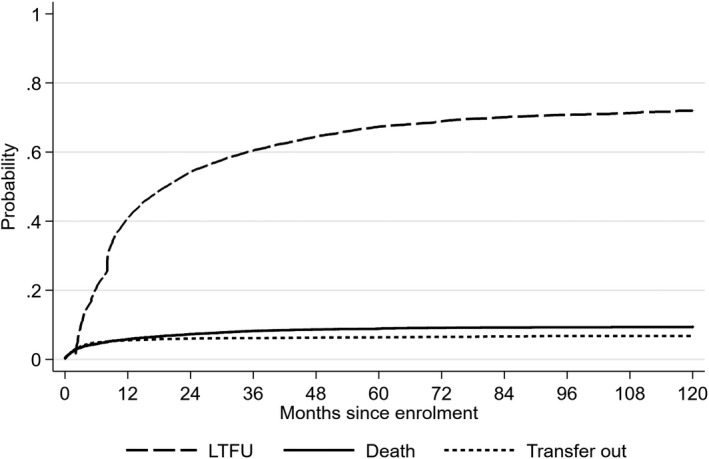
Cumulative incidence function for the first event. LTFU, lost to follow‐up.

The median follow‐up time was 10 months (interquartile range, IQR 5 to 26). The cumulative incidence of LTFU at one year was 0.41 (95% confidence interval, CI 0.40 0.42) and 0.67 (0.66, 0.68) at five years (Figure 1). The cumulative incidences of death and transfer were much lower, both at 0.06 (0.05, 0.06) at one year and remaining fairly steady thereafter.

LTFU was associated with being male, younger age, never married, and living far from the clinic (Table 2). Underweight participants and those with more advanced WHO stage had increased risk of being LTFU, and there was some suggestion that those with higher CD4 counts were less likely to be LTFU. Factors which were associated with lower LTFU risk were having an HIV‐positive partner, having tuberculosis, being pregnant, and longer time since ART initiation. Participants enrolled in 2005 to 2007 and 2013 to 2014 were at lowest LTFU risk. There was no evidence of an interaction between CD4 count and enrolment year (*p* = 0.30).

**Table 2:** Factors associated with lost to follow‐up

Characteristic	Cause‐specific proportional hazard model; first event HR (95% CI)	Cause‐specific proportional hazard model; last event HR (95% CI)	Marginal means/rates model; recurrent events RR (95% CI)	PWP‐total model; recurrent events HR (95% CI)	PWP‐gap model; recurrent events HR (95% CI)
Sex					
Male versus female	1.24 (1.16, 1.32)	1.27 (1.17, 1.37)	1.17 (1.11, 1.23)	1.18 (1.12, 1.25)	1.23 (1.17, 1.29)
Age, years					
15 to 24	1.20 (1.07, 1.34)	1.10 (0.96, 1.27)	1.15 (1.04, 1.27)	1.15 (1.05, 1.26)	1.18 (1.08, 1.29)
25 to 34	1.12 (1.04, 1.20)	1.08 (0.99, 1.18)	1.06 (1.00, 1.13)	1.07 (1.01, 1.13)	1.09 (1.03, 1.15)
35 to 44	Ref	Ref	Ref	Ref	Ref
≥45	0.92 (0.86, 0.99)	0.97 (0.88, 1.06)	0.96 (0.89, 1.02)	0.96 (0.90, 1.02)	0.94 (0.89, 1.00)
Marital status					
Married/cohabiting	Ref	Ref	Ref	Ref	Ref
Never married	1.23 (1.12, 1.35)	1.65 (1.49, 1.83)	1.28 (1.19, 1.38)	1.31 (1.21, 1.41)	1.37 (1.28, 1.47)
Separated/divorced/widowed	0.94 (0.88, 1.01)	0.89 (0.81, 0.98)	0.97 (0.92, 1.03)	0.97 (0.92, 1.03)	0.97 (0.92, 1.03)
Distance of residence from clinic, km					
≤1 (i.e. resident in Ifakara town)	Ref	Ref	Ref	Ref	Ref
2 to <50	0.85 (0.79, 0.92)	0.86 (0.78, 0.95)	0.92 (0.86, 0.98)	0.91 (0.86, 0.97)	0.88 (0.83, 0.93)
50 to <80	1.22 (1.11, 1.35)	1.68 (1.51, 1.88)	1.36 (1.26, 1.47)	1.34 (1.24, 1.44)	1.34 (1.24, 1.45)
≥80	1.26 (1.16, 1.37)	1.31 (1.18, 1.45)	1.30 (1.22, 1.39)	1.31 (1.23, 1.40)	1.42 (1.33, 1.51)
Partner HIV sero‐status					
Positive	0.82 (0.74, 0.90)	0.78 (0.69, 0.89)	0.86 (0.79, 0.93)	0.87 (0.80, 0.94)	0.85 (0.79, 0.91)
Negative	0.92 (0.82, 1.04)	0.97 (0.84, 1.13)	1.00 (0.90, 1.11)	0.99 (0.90, 1.08)	0.96 (0.87, 1.06)
Unknown	Ref	Ref	Ref	Ref	Ref
Not applicable	0.93 (0.81, 1.08)	0.89 (0.75, 1.06)	0.99 (0.87, 1.13)	0.99 (0.88, 1.11)	0.99 (0.88, 1.11)
HIV disclosure status					
Disclosed versus not disclosed	0.96 (0.89, 1.04)	0.90 (0.82, 0.98)	0.95 (0.89, 1.01)	0.95 (0.89, 1.00)	0.98 (0.93, 1.04)
BMI, kg/m^2^					
Underweight (<18.5)	1.15 (1.07, 1.25)	1.27 (1.16, 1.39)	1.07 (1.00, 1.13)	1.07 (1.01, 1.14)	1.10 (1.04, 1.17)
Normal (18.5 to <25)	Ref	Ref	Ref	Ref	Ref
Overweight (≥25)	0.93 (0.84, 1.02)	0.82 (0.72, 0.93)	0.94 (0.86, 1.02)	0.95 (0.87, 1.03)	0.94 (0.87, 1.01)
CD4 count, cells/mm^3^					
<100	Ref	Ref	Ref	Ref	Ref
100 to 199	1.00 (0.88, 1.13)	0.92 (0.81, 1.04)	0.99 (0.88, 1.11)	1.00 (0.90, 1.12)	1.02 (0.89, 1.17)
200 to 349	1.00 (0.88, 1.13)	0.95 (0.83, 1.08)	1.00 (0.91, 1.09)	1.01 (0.93, 1.11)	1.00 (0.90, 1.12)
350 to 499	0.86 (0.74, 0.99)	0.84 (0.72, 0.98)	0.96 (0.85, 1.08)	0.96 (0.84, 1.09)	0.93 (0.84, 1.04)
≥500	0.86 (0.76, 0.99)	0.88 (0.76, 1.03)	1.01 (0.91, 1.12)	0.97 (0.86, 1.09)	0.92 (0.83, 1.02)
WHO stage					
Stage 1	Ref	Ref	Ref	Ref	Ref
Stage 2	0.97 (0.90, 1.05)	0.92 (0.83, 1.02)	0.93 (0.87, 1.00)	0.92 (0.86, 0.98)	0.95 (0.89, 1.01)
Stage 3	1.16 (1.07, 1.25)	1.17 (1.05, 1.29)	1.08 (1.00, 1.15)	1.07 (1.00, 1.15)	1.14 (1.07, 1.21)
Stage 4	1.34 (1.20, 1.49)	1.61 (1.43, 1.82)	1.26 (1.15, 1.37)	1.28 (1.18, 1.39)	1.35 (1.24, 1.46)
Tuberculosis status					
Positive versus negative	0.86 (0.75, 0.99)	0.80 (0.68, 0.95)	0.86 (0.76, 0.97)	0.84 (0.75, 0.95)	0.87 (0.78, 0.98)
Time since ART initiation, months					
Not yet initiated ART	Ref	Ref	Ref	Ref	Ref
0 to <6	0.81 (0.75, 0.89)	0.61 (0.55, 0.68)	0.60 (0.56, 0.65)	0.55 (0.51, 0.59)	0.55 (0.51, 0.60)
6 to <12	0.26 (0.24, 0.29)	0.32 (0.27, 0.37)	0.67 (0.62, 0.73)	0.60 (0.55, 0.66)	0.54 (0.49, 0.59)
12 to <24	0.23 (0.20, 0.25)	0.47 (0.40, 0.55)	0.60 (0.56, 0.65)	0.57 (0.52, 0.61)	0.37 (0.34, 0.40)
≥24	0.13 (0.12, 0.15)	0.36 (0.31, 0.42)	0.50 (0.46, 0.55)	0.47 (0.43, 0.51)	0.32 (0.30, 0.35)
Pregnant					
Yes versus no	0.70 (0.58, 0.84)	1.54 (1.20, 1.98)	1.27 (1.08, 1.49)	1.25 (1.07, 1.46)	1.69 (1.44, 1.98)
Year of registration					
2005 to 2007	0.53 (0.49, 0.58)	0.71 (0.64, 0.78)	0.74 (0.68, 0.80)	0.79 (0.74, 0.86)	0.69 (0.65, 0.74)
2008 to 2009	Ref	Ref	Ref	Ref	Ref
2010 to 2012	0.96 (0.89, 1.04)	1.25 (1.12, 1.38)	1.32 (1.22, 1.44)	1.38 (1.28, 1.50)	0.91 (0.85, 0.97)
2013 to 2014	0.59 (0.52, 0.66)	0.76 (0.64, 0.89)	1.32 (1.16, 1.51)	1.88 (1.65, 2.14)	0.60 (0.54, 0.66)
2015 to 2016	0.83 (0.73, 0.94)	2.00 (1.73, 2.32)	2.18 (1.88, 2.53)	3.31 (2.82, 3.87)	0.94 (0.85, 1.05)

Results are from models with multiple imputation for missing baseline covariates. All variables are defined at baseline, except time since ART initiation and pregnancy which are time‐dependent. ART, antiretroviral therapy; BMI, body mass index; CI, confidence interval; HR, hazard ratio; RR, rate ratio.

### 3.2 Last event

Compared to the analysis based on the first event, under the last event analysis the number of participants LTFU was much smaller (3110, 38%), the number in active care was higher (3203, 40%), and deaths and transfers out were broadly similar (811, 10% and 963, 12% respectively). Characteristic distributions by last event were similar to those by first event. The cumulative incidence of LTFU at one year was 0.21 (95% CI 0.20, 0.22) and 0.38 (0.37, 0.39) at five years (Figure S1). The corresponding results for death were 0.06 (0.06, 0.07) and 0.10 (0.09, 0.10), and for transfers 0.05 (0.04, 0.05) and 0.12 (0.11, 0.13).

The factors associated with LTFU were broadly similar to the first event analysis, except that there was no longer as strong a relationship with age, disclosure of HIV status was associated with lower LTFU risk, there was a stronger trend towards higher LTFU risk with lower BMI, and pregnancy was associated with higher risk of LTFU (Table 2). In addition, participants enrolled in 2010 to 2012 and particularly 2015 to 2016 were at higher risk of LTFU versus 2008 to 2009.

### 3.3 Recurrent LTFU events

**Figure 2:**
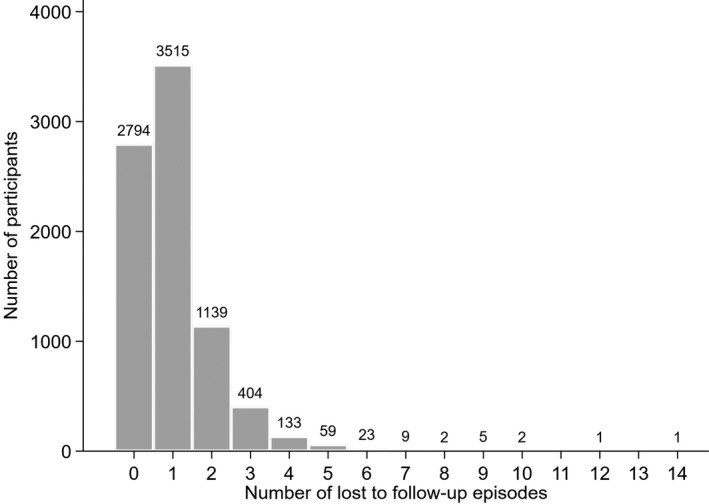
Distribution of lost to follow‐up events. Participants who remained in care, died or transferred out without ever being lost to follow‐up are included as having zero lost to follow‐up events. For the Prentice, Williams and Peterson (PWP) models, the number of events was truncated at four.

There were 8140 LTFU episodes, following which there were 2483 (31%) returns to care after a median of three months following the last scheduled appointment (IQR 2 to 6). Overall, 5293 (65%) participants had at least one LTFU episode, with a median of one episode/participant (Figure 2). There were 1387 transfers out among 1360 (17%) participants, 811 (10%) died and 1781 (22%) remained in care throughout. Among the 5105 participants whose first event was LTFU, 2583 (51%) returned to care. Participant characteristics were broadly similar among those who did versus did not return to care, except that those who did not return tended to be more likely to have never married, be underweight, have more advanced WHO stage, have lower CD4 count, and have shorter time from enrolment to LTFU (Table S1). Over all follow‐up, 6187 (77%) participants initiated ART and there were 1351 pregnancies in 998 (19%) women.

Associations between LTFU and covariates in the recurrent events models were broadly similar to the first LTFU event analysis, except that there was no longer evidence of an association with CD4 count, and pregnancy was associated with higher risk of LTFU (Table 2). In addition, there were differences in the pattern of recurrent LTFU by enrolment year under the marginal means/rates and PWP‐total models, with those enrolled in later years at higher risk of recurrent LTFU. Under the PWP‐gap model, the relationship between recurrent LTFU and enrolment year was broadly similar to that in the first event analysis. There was no evidence of interactions between CD4 count and enrolment year (*p* = 0.97, 0.97 and 0.42 for the marginal means/rates, PWP‐total and PWP‐gap models respectively).

## 4 Discussion

In this large, rural cohort of adults living with HIV, the probability of LTFU was high when analysed prospectively, at 41% by one year after enrolment and stabilizing around 70% by five years. However, transient interruptions in care were common, with a third of LTFU episodes followed by a return to care, and overall two‐thirds of participants experiencing at least one LTFU episode. Considering the last event before database closure, the probability of LTFU at one year was roughly half, at 21%, similar to a previous study in Tanzania 13. The large difference between cumulative incidences based on the first and last event definitions has been noted by others, with the last event approach tending to overestimate LTFU in later years 11. While the different approaches represent different research questions, our results illustrate the importance of clear definitions and exercising caution in comparing estimates across studies. Studies assessing LTFU predictors should use a prospective definition of LTFU, and take into account period of enrolment 11. Retrospective outcome definitions may be appropriate in other situations, for example in mortality assessment, since it allows for the inclusion of more follow‐up time 11.

Participant characteristics associated with LTFU can be used to identify higher risk individuals for tracing after non‐attendance and to help design appropriate interventions to improve retention in care. Our findings are in line with the literature: young people are more difficult to retain in long‐term care because they are more likely to be mobile while searching for employment opportunities 41, 42. Participants who were never married and those without HIV‐positive partners were at higher LTFU risk, which may be due to a lack of family support 43, 44 and/or stigma 45. Furthermore, the high incidence of LTFU during the first year may be linked to stigma 46, suggesting that counselling and support following HIV diagnosis may help patients to accept their diagnosis and increase motivation to continue with life‐long HIV treatment 46. A substantial proportion of participants in our study lived far from the clinic (31% >50 km), and this was associated with a higher LTFU risk. Accessibility problems such as lack of transport, cost or distance have previously been identified as barriers to clinic attendance 43, 44. Potential interventions could include longer ART refills for those stable on treatment as recently recommended by the Tanzanian government 47, 48, decentralization of care to local health clinics, or alternative ART delivery models such as to the home or through the community 49. Participants with low BMI or advanced WHO stage were more likely to become LTFU, probably indicating disease progression with unreported deaths 50. Furthermore, being sick may directly impair clinic attendance 44. Participants with tuberculosis were less likely to become LTFU, probably related to their existing engagement in care for another chronic condition. The high incidence of LTFU among ART‐naïve participants in our cohort has been observed in previous studies, albeit using different LTFU definitions 41, 43, 51. Historically, ART was not initiated until clinically indicated, and therefore individuals did not need to attend the clinic to collect refills 26, 27, 28, 29. With recent changes in guidelines to recommend treatment for all, this inherent bias may be ameliorated 52. Regardless, two‐thirds of LTFU episodes occurred in participants on ART, likely resulting in suboptimal adherence and therefore risk of poorer individual outcomes 53, 54 and onwards sexual transmission 55, 56. However, such participants may have sought drug refills from another clinic without our knowledge. Regardless of ART status, participants who were LTFU from our clinic may have “silently” transferred care to another clinic 57 or died without notification to the clinic 50, particularly given that those participants who were LTFU and did not return to the clinic had poorer clinical prognoses than those who returned. The national electronic database of HIV care and treatment centres does not currently allow extraction of data from other centres, which would help to trace patients LTFU. Future studies could investigate the impact of silent transfers and unreported deaths through such linkage with other clinics and/or tracing in the community.

The models for assessing first and recurrent LTFU events have different underlying assumptions, estimate different parameters, and hence have different interpretations. Despite these differences, we observed comparable results across the models, suggesting that in this population the factors associated with first, last and recurrent LTFU were broadly similar. One exception was pregnancy which was associated with lower LTFU risk in the first event analysis, but higher risk in the recurrent and last event models, in line with previous studies 20, 21. This highlights the higher risk of recurrent LTFU among pregnant women who have already struggled with attendance. The second exception was CD4 count: higher baseline levels were associated with lower LTFU in the first event analysis, but not in the recurrent or last event analyses, likely due to unreported deaths in the short‐term among participants with low baseline values. Lastly, there was a trend towards higher LTFU risk in later enrolment years under the last event analysis, but this is biased due to transient interruptions in care 11. We observed similar trends when modelling recurrent LTFU events with marginal means/rates and PWP‐total models, but under the PWP‐gap model and for the first event analysis we observed somewhat lower LTFU risks in later years. With changes in HIV guidelines, which can influence HIV care and treatment service delivery and hence modify participants’ behaviour, improvement of data collection tools in our clinic, and increased awareness about HIV in the community, we would anticipate the incidence of LTFU to decrease over time. Further research is required to determine whether the marginal means/rates and PWP‐total models may be susceptible to the same biases as the last event analyses. Some bias may remain in the estimates from the recurrent event models if long periods are permitted before declaring a participant LTFU 11; we chose a delay of 60 days based on the recommendations of previous studies 10, 24. Furthermore, participants were not at risk for further events during a LTFU episode (which may have long duration). An alternative approach would be to use multi‐state models, which explicitly model the probabilities of transitioning between different states, for example LTFU, under care, transfer to other clinics or death 12.

Strengths of our study include the long cohort follow‐up, the standardized data capture system, and use of multiple imputation to address missing baseline covariates. Furthermore, we appropriately addressed recurrent LTFU episodes which are often neglected or incompletely described in many studies. The study has several limitations. Pregnancy was recorded only as a binary status at each visit; capture of delivery dates would enable us to better examine the pregnancy and postpartum periods. We included as a covariate the time since ART initiation, with the assumption that once individuals initiate ART then they remain on it. The multiple imputation methods assume that data were missing at random; we cannot assess this assumption in the data but we increased the plausibility of our findings by incorporating a broad range of covariates 35. Education and occupation were not collected pre‐2013 and therefore were not included in the multivariable analyses, but may mediate the effect of some of the other covariates modelled. We considered it important to apply a consistent LTFU definition throughout the cohort, but visits were scheduled approximately monthly pre‐2012 and for pregnant women, therefore we may have underestimated LTFU incidence in some participants.

## 5 Conclusions

In this cohort, LTFU episodes were common, potentially jeopardizing the success of ART for individual prognosis and treatment as prevention 2, 3. Optimization of retention in care must be prioritized if we are to improve the long‐term outcomes of HIV‐positive persons and reach the third of the UNAIDS 90‐90‐90 targets to curb the HIV epidemic 2. We have identified important socio‐demographic and clinical characteristics which may be used to target prompt tracing efforts or inform the design of interventions, such as longer ART refills or home/community ART delivery for stable patients living far from the clinic, and connecting those without sufficient social support with treatment supporters. Furthermore, we have demonstrated the effects of transient interruptions in care on the incidence of and risk factors for LTFU, and the importance of clearly describing and appropriately accounting for such episodes. We recommend using a prospective definition of LTFU combined with recurrent event analyses in cohorts where repeated interruptions in care are common.

## Competing interests

All authors declare no conflicts of interest.

## Authors’ contributions

AVK, TRG, MW and FV designed the study. MW, SJM, BK, YK, GS and AK contributed to data collation. AVK and FV performed the statistical analyses. TRG, MW, MB and FV supervised the work. AVK and FV wrote the first draft of the manuscript. All authors reviewed and approved the manuscript.

## Supporting information


**Table S1.** Characteristics by whether returned to care following a first LTFU episode, among participants whose first event was LTFU.
**Figure S1.** Cumulative incidence function for the last event captured at database closure.
