# Supplementary material for: Prospective assessment of loss to follow‐up: incidence and associated factors in a cohort of HIV‐positive adults in rural Tanzania
Source: J Int AIDS Soc. 2020 Mar 3;23(3):e25460. doi: 10.1002/jia2.25460 (PMC7054631; doi:10.1002/jia2.25460)
Supplement: Supplementary file 1 — Table S1. Characteristics by whether returned to care following a first LTFU episode, among participants whose first event was LTFU. Figure S1. Cumulative incidence function for the last event captured at database closure. [file JIA2-23-e25460-s001.docx]

**SUPPORTING INFORMATION**

**Table S1. Characteristics by whether returned to care following a first LTFU episode, among participants whose first event was LTFU.**

| **Characteristic** | **Did not return** | **Returned** | **Total with first event LTFU** |
| --- | --- | --- | --- |
| Total, number (row %) | 2522 (49%) | 2583 (51%) | 5105 (100%) |
| **Baseline characteristics** |  |  |  |
| Sex |  |  |  |
| Male | 983 (39%) | 858 (33%) | 1841 (36%) |
| Female | 1539 (61%) | 1710 (67%) | 3249 (64%) |
| *Missing* | *0* | *15 (1%)* | *15 (<1%)* |
| Age, years |  |  |  |
| 15-24 | 223 (9%) | 195 (8%) | 418 (8%) |
| 25-34 | 872 (35%) | 851 (33%) | 1723 (34%) |
| 35-44 | 835 (33%) | 916 (35%) | 1751 (34%) |
| ≥45 | 592 (23%) | 621 (24%) | 1213 (24%) |
| Marital status |  |  |  |
| Married/ cohabiting | 1281 (52%) | 1390 (55%) | 2671 (53%) |
| Never married | 532 (22%) | 310 (12%) | 842 (17%) |
| Separated/ divorced/ widowed | 640 (26%) | 845 (33%) | 1485 (30%) |
| *Missing* | *69 (3%)* | *38 (1%)* | *107 (2%)* |
| Distance of residence from clinic, km |  |  |  |
| ≤1 (i.e. resident in Ifakara town) | 880 (42%) | 1055 (44%) | 1935 (43%) |
| 2-<50 | 447 (21%) | 537 (23%) | 984 (22%) |
| 50-<80 | 343 (16%) | 262 (11%) | 605 (13%) |
| ≥80 | 439 (21%) | 521 (22%) | 960 (21%) |
| *Missing* | *413 (16%)* | *208 (8%)* | *621 (12%)* |
| Partner HIV sero-status |  |  |  |
| Positive | 269 (11%) | 367 (15%) | 636 (13%) |
| Negative | 162 (7%) | 176 (7%) | 338 (7%) |
| Unknown | 1807 (74%) | 1798 (72%) | 3605 (73%) |
| Not applicable | 192 (8%) | 148 (6%) | 340 (7%) |
| *Missing* | *92 (4%)* | *94 (4%)* | *186 (4%)* |
| HIV status disclosure |  |  |  |
| Not disclosed | 756 (37%) | 730 (34%) | 1486 (35%) |
| Disclosed | 1292 (63%) | 1444 (66%) | 2736 (65%) |
| *Missing* | *474 (19%)* | *409 (16%)* | *883 (17%)* |
| Pregnant* |  |  |  |
| No | 1463 (95%) | 1634 (96%) | 3097 (95%) |
| Yes | 76 (5%) | 76 (4%) | 152 (5%) |
| BMI, kg/m^2^** |  |  |  |
| Underweight (<18.5) | 550 (30%) | 527 (24%) | 1077 (27%) |
| Normal (18.5-<25) | 1089 (59%) | 1325 (60%) | 2414 (60%) |
| Overweight (≥25) | 215 (12%) | 341 (16%) | 556 (14%) |
| *Missing* | *592 (24%)* | *314 (13%)* | *906 (18%)* |
| CD4 count, cells/mm^3^ |  |  |  |
| <100 | 403 (28%) | 338 (23%) | 741 (25%) |
| 100-199 | 243 (17%) | 311 (21%) | 554 (19%) |
| 200-349 | 290 (20%) | 319 (21%) | 609 (21%) |
| 350-499 | 198 (14%) | 231 (16%) | 429 (15%) |
| ≥500 | 294 (21%) | 291 (20%) | 585 (20%) |
| *Missing* | *1094 (43%)* | *1093 (42%)* | *2187 (43%)* |
| WHO stage |  |  |  |
| Stage 1 | 936 (38%) | 1052 (42%) | 1988 (40%) |
| Stage 2 | 442 (18%) | 577 (23%) | 1019 (21%) |
| Stage 3 | 673 (28%) | 626 (25%) | 1299 (26%) |
| Stage 4 | 383 (16%) | 235 (9%) | 618 (13%) |
| *Missing* | *88 (3%)* | *93 (4%)* | *181 (4%)* |
| Tuberculosis status |  |  |  |
| No | 2335 (94%) | 2412 (96%) | 4747 (95%) |
| Yes | 143 (6%) | 100 (4%) | 243 (5%) |
| *Missing* | *44 (2%)* | *71 (3%)* | *115 (2%)* |
| ART status (within 30 days of enrolment) |  |  |  |
| Not yet initiated ART | 1615 (64%) | 1557 (60%) | 3172 (62%) |
| Initiated ART | 907 (36%) | 1026 (40%) | 1933 (38%) |
| Year of registration |  |  |  |
| 2005-07 | 665 (26%) | 647 (25%) | 1312 (26%) |
| 2008-09 | 794 (31%) | 982 (38%) | 1776 (35%) |
| 2010-12 | 531 (21%) | 538 (21%) | 1069 (21%) |
| 2013-14 | 173 (7%) | 288 (11%) | 461 (9%) |
| 2015-16 | 359 (14%) | 128 (5%) | 487 (10%) |
| **Characteristics at first LTFU episode** |  |  |  |
| Time to first LTFU episode, months (median and interquartile range) | 8 (4-12) | 11 (5-23) | 8 (5-18) |
| Last CD4 count, cells/mm^3^ *** |  |  |  |
| <100 | 346 (20%) | 204 (10%) | 550 (15%) |
| 100-199 | 270 (16%) | 281 (14%) | 551 (15%) |
| 200-349 | 361 (21%) | 515 (26%) | 876 (24%) |
| 350-499 | 289 (17%) | 429 (21%) | 718 (19%) |
| ≥500 | 422 (25%) | 589 (29%) | 1011 (27%) |
| *Missing* | *834 (33%)* | *565 (22%)* | *1399 (27%)* |
| Last WHO stage^a^ |  |  |  |
| Stage 1 | 804 (33%) | 828 (33%) | 1632 (33%) |
| Stage 2 | 397 (16%) | 588 (24%) | 985 (20%) |
| Stage 3 | 727 (30%) | 724 (29%) | 1451 (29%) |
| Stage 4 | 510 (21%) | 344 (14%) | 854 (17%) |
| *Missing* | *84 (3%)* | *99 (4%)* | *183 (4%)* |

Results are number and column % of those with non-missing data, unless otherwise indicated; missing data rows are number and column %. ART=antiretroviral therapy. BMI=body mass index. *Percentages are of number of women. **Excluding pregnant women. ***Last measurements before first LTFU episode.

**Figure S1. Cumulative incidence function for the last event captured at database closure.**

LTFU=lost to follow-up.
